# High-Resolution UPLC-MS Profiling of Anthocyanins and Flavonols of Red Cabbage (*Brassica oleracea* L. var. *capitata* f. *rubra* DC.) Cultivated in Egypt and Evaluation of Their Biological Activity

**DOI:** 10.3390/molecules26247567

**Published:** 2021-12-14

**Authors:** Khaled Ahmed Mansour, Sherifa Fahmy Moustafa, Soad Mohamed Abdelkhalik

**Affiliations:** 1 Pharmacognosy Department, Faculty of Pharmacy, October 6 University, Al Mehwar Al Markazi, Giza 12585, Egypt; sherifa.mostafa@pharma.cu.edu.eg; 2 Pharmacognosy Department, Faculty of Pharmacy, Horus University in Egypt, New Damietta 34517, Egypt; 3 Pharmacognosy Department, Faculty of Pharmacy, Cairo University, Kasr el Aini st., Cairo 11562, Egypt; 4 Pharmacognosy Department, Faculty of Pharmacy, Helwan University, Ain-Helwan, Cairo 11795, Egypt; soad_abdelkhalik@pharm.helwan.edu.eg

**Keywords:** high-resolution mass, anthocyanins, antioxidant, antimicrobial, anticancer, statistical significance

## Abstract

In this paper, biological investigations and a high-resolution UPLC-PDA-ESI-qTOF-HRMS technique were employed for *Brassica oleracea* L. var. *capitata* f. *rubra* DC. (red cabbage) of the family Brassicaceae (Cruciferae), cultivated in Egypt, for the first time. The positive ionization mode is usually performed to identify anthocyanins. However, this technique cannot differentiate between anthocyanins and corresponding non-anthocyanin polyphenols. Thus, the negative ionization mode was also used, as it provided a series of characteristic ions for the MS analysis of anthocyanins. This helped in identifying five kaempferol derivatives for the first time in red cabbage, as well as nine—previously reported—anthocyanins. For the biological investigations, the acidified methanolic extract of fresh leaves and the methanolic extract of air-dried powdered leaves were examined for their antioxidant, antimicrobial, and anticancer activities. The freshly prepared phenolic extract was proven to be more biologically potent. Statistical significance was determined for its anticancer activity in comparison with standard doxorubicin.

## 1. Introduction

Anthocyanins are brightly colored pigments that have attracted the interest of many researchers in recent decades. They are found in different natural sources (fruits and vegetables) which may vary in color and/or composition. Unlike the anthocyanins present in different fruits and vegetables, the anthocyanins of red cabbage leaves are highly conjugated with a variety of sugars and/or acyl groups; with the lack of pure standards, it would be a challenge to fulfill their characterization and identification [1,2,3,4]. Several studies on the characterization and identification of secondary metabolites in complex plant extracts showed that liquid chromatography coupled to photodiode array detection is very effective for anthocyanins analysis [5,6].

At an acidic pH, anthocyanins exist in a positively charged cationic form; hence, the positive ionization mode has typically been used for the mass analysis of anthocyanins. However, anthocyanins cannot be distinguished from corresponding flavonol glycosides in the positive ionization mode using mass spectrometry. In negative ionization mode, a series of ions, e.g., [M − 2H + H_2_O]^–^ and [M − 2H]^−^ doublet ions, were found to be characteristic for the MS analysis of anthocyanins [7]. Therefore, the negative ionization mode was also used in this analysis.

The anthocyanins profiling of the leaves of *Brassica oleracea* L. var. *capitata* f. *rubra* growing in Sweden, the United States, and China was previously investigated [1,8,9,10]. All the previously reported anthocyanin aglycones were cyanidin, glycosylated mainly with glucose and/or sophorose (diglucoside), and acylated with various aromatic and aliphatic acids (caffeoyl, *p*-coumaroyl, feruloyl, *p*-hydroxybenzoyl, sinapoyl, and oxaloyl). Red cabbage growing in India, China, and many other countries was investigated for its antioxidant, antimicrobial, and anticancer activities [11,12,13]. On the contrary, nothing was traced concerning the anthocyanins content and/or the biological effects of red cabbage cultivated in Egypt.

Considering the increasing numbers of cancer victims in Egypt, especially those not yet in their twenties [14], and because of the numerous side effects of the current therapies, it was necessary to find an alternative natural remedy with minimal (or hopefully with no) side effects. Being promising natural pharmaceuticals, it was of interest to define the phytochemical composition of the anthocyanins content in Egyptian red cabbage leaves, as well as the co-existing phenolics, using high-resolution (UPLC-MS) profiling, and to examine its pharmacological activity as an anticancer, antioxidant, and antimicrobial agent in attempt to use it as a natural original remedy in such fields.

The methanolic extract of air-dried powdered leaves as well as the acidified methanolic extract of fresh leaves of red cabbage were investigated for their antioxidant, antimicrobial, and anticancer biological activities. The antioxidant activity was measured using the ferric reducing antioxidant power (FRAP) method and the antimicrobial activity was determined using the agar well diffusion assay. Minimum inhibitory concentration (MIC) was also determined against the examined organisms. The anticancer activity was determined using a sulforhodamine-B (SRB) assay and a statistical significance analysis was performed in comparison with standard doxorubicin.

## 2. Materials and Methods

### 2.1. Plant Material

Three heads of *Brassica oleracea* L. var. *capitata* f. *rubra* DC (red cabbage) were obtained at full maturity in July 2016 from Nubaria farms (30°40′16.428″ N 30°4′33.024″ E), Alex-Cairo desert highway after permission was obtained from the landowner. Plant collection complied with relevant national and international guidelines and legislation. Identification was performed through the courtesy of Dr. A. Abd-Elmogali, a specialized taxonomist at the Agricultural Research Centre, Giza, Egypt and a voucher specimen (voucher code: BOCR375) was deposited in the herbarium of the department of Pharmacognosy, Faculty of Pharmacy, October 6 University, Giza, Egypt.

### 2.2. Preparation of the Extracts

The total alcoholic extract of *B. oleracea* L. var. *capitata* f. *rubra* DC was prepared by the classical method; air-dried powdered leaves (100 g) were extracted using methanol (cold maceration) until exhaustion. The solvent was then removed by vacuum distillation at a temperature not exceeding 40 °C and the residue was saved for biological investigations.

On the other hand, fresh leaves were used for preparing the phenolic extract. Fresh leaves (100 g) were cut using a knife into small pieces (5–7 mm), and then macerated in 1 L of methanol/HCl (98:2 *v*/*v*) for 24 h at 4 °C. The powder was extracted until exhaustion by repeating the process of extraction under the same conditions 3 times. The acidified methanolic extracts were combined, filtered, freeze-dried, and separated into two parts, one for the UPLC-MS analysis and the other for the biological investigation. All experiments involving plants adhered to relevant ethical guidelines.

For UPLC-MS analysis, 25 mg of the freeze-dried phenolic extract was homogenized with 2.5 mL of 70% MeOH containing 5 µg/mL of umbelliferone (as the internal standard for relative quantification using UPLC–MS) using a Turrax mixer (11,000 RPM) for five 20 s periods. Each mixing period was separated by a cooling period of 1 min to prevent heating. The extract was then vortexed vigorously and centrifuged at 3000× *g* for 30 min to remove plant debris. Then, 500 µL was aliquoted and placed on a (500 mg) C18 cartridge which was preconditioned with methanol and water. Samples were eluted using 3 mL of 70% MeOH and 3 mL of 100% MeOH. The eluent was evaporated under a nitrogen stream and the obtained dry residue was resuspended in 500 µL of methanol. Three microliters were used for analysis.

### 2.3. High-Resolution UPLC-MS Analysis

Chromatographic separation was performed on an Acquity UPLC system (Waters, Milford, MA, USA) equipped with a HSS T3 column (100 × 1.00 mm, particle size 1.8 µm; Waters). The analysis was done using the following binary gradient at flow rate of 150 µL min^−1^: 0 to 1 min, isocratic 95% A (water/formic acid, 99.9/0.1 (*v*/*v*)), 5% B (acetonitrile/formic acid, 99.9/0.1 (*v*/*v*)); 1 to 16 min, linear from 5 to 95% B; 16 to 18 min, isocratic 95% B; and 18 to 20 min, isocratic 5% B.

The injection volume was 3.1 µL (full loop injection). Eluted compounds were detected *m*/*z* 0 to 1000 using these instrument settings: nebulizer gas, nitrogen, 1.6 bar; dry gas, nitrogen, 6 L min^−1^, 190 °C; capillary, −5500 V (+4000 V); end plate offset, −500 V; hexapole RF, 100 Vpp; funnel 2 RF, 200 Vpp; in-source CID energy, 0 V; hexapole RF, 100 Vpp; quadrupole ion energy, 5 eV; collision gas, argon; collision energy, 10 eV; collision RF, 200/400 Vpp (timing 50/50); transfer time, 70 µs; prepulse storage, 5 µs; pulser frequency, 10 kHz; and spectra rate, 3 Hz. Internal mass calibration of each analysis was performed by an infusion of 20 µL of 10 mM lithium formate in isopropanol:water, 1:1 (*v*/*v*), at a gradient time of 18 min using a diverter valve.

Tentative identification was done by comparing the retention time, UV-vis spectra and high-resolution MS spectrometry (accurate mass and MS/MS fragmentation patterns) of the compounds detected with the reported data [1,8,9,10] and searching in the existing phytochemical dictionary of natural products database (Compact Reinforced Composite (CRC), Wiley).

### 2.4. Antioxidant Determination

The total antioxidant capacity (TAC) of both extracts was determined using the ferric reducing antioxidant power (FRAP) method. The extracts were prepared in a concentration of 50 μg/mL. Ferrous sulfate heptahydrate (FeSO_4_·7H_2_O) was purchased from Sigma-Aldrich (St. Louis, MO, USA) and was used as a standard at a concentration of 1000 µM [15]. Antioxidant activity was expressed as the concentration of antioxidants with a ferric reducing ability equivalent to that of 1 mM/L FeSO_4_·7H_2_O.

### 2.5. Antimicrobial Determination

The antimicrobial activity was determined using the agar well diffusion assay. The extracts and all the standards were prepared as 100 μg/mL. Sterile plates were inoculated with the fresh cultures of *Staphylococcus aureus* ATCC 6538, *Bacillus subtilis* ATCC 6633, *Candida albicans* ATCC 10231, *Escherichia coli* ATCC 8739, *Pseudomonas aeruginosa* ATCC 9027, and *Aspergillus niger* ATCC 16404, obtained from the microbiology laboratory of the Faculty of Pharmacy, October 6 University, Giza, Egypt. Antimicrobial activity was determined by measuring the zones of inhibition (in mm) using ampicillin as a standard against gm +ve bacteria, streptomycin as a standard against gm −ve bacteria, and clotrimazole as an antifungal standard [16]; all standards were purchased from Sigma-Aldrich, Germany. The minimum inhibitory concentrations (MIC) were estimated against each of the tested organisms in triplicate.

### 2.6. Anticancer Determination

The cytotoxicity investigation of the two extracts was carried out using a sulforhodamine-B (SRB) assay following the method reported by [17]. The potential cytotoxicity was determined against HeLa, MCF-7, and HepG-2 cell lines obtained from the National Research Centre, Giza, Egypt, using doxorubicin as a standard. Doxorubicin was supplied from Pharmacia of Upjohn S.P.A. Research. All samples were prepared by dissolving in dimethylsulfoxide (DMSO) at 100 mM and were stored at −20 °C.

### 2.7. Statistical Analysis

All statistical analyses were performed using GraphPad Prism version 9.2.0 to calculate IC_50_ and the level of significance was set at *p* > 0.05. Quantitative data were described as mean ± standard deviation (SD). GraphPad Prism version 9.2.0 was also used to create multiple bar charts of the cytotoxic activity and cell viability.

## 3. Results and Discussion

### 3.1. Identification of Compounds

(Arapitsas et al. 2008) used the peak spectral characteristic λ_vis_ (at 520 nm), λ_440_ (at 440 nm), λ_acyl_ (at 286 nm), and their corresponding absorptivities to identify the mono- or bioside anthocyanins (E_440_/E_vis_ absorptivity ratio of 29–35% for a monoside and 15–24% for a bioside) and E_acyl_/E_vis_ absorptivity ratio to determine the degree of aromatic acylation (53–69% for monoacylation and 98–128% for diacylation). However, these reported absorptivity ratios are not absolute and vary according to the extraction and analysis conditions used. This is because the spectral characteristics of anthocyanins strongly depend on pH, and dramatically change using different conditions and/or solvents for extraction [18,19,20,21].

In the present study, both positive (Figure S1 in Supplementary Material) and negative (Figure S2) ionization modes were performed. The interpretation of the high resolution UPLC-MS data of all the identified compounds was carried out depending on the previously reported data and the phytochemical dictionary of natural products database.

Nine anthocyanins (A_1_–A_9_) and five phenolics (K_1_–K_5_) were identified in the leaves of red cabbage cultivated in Egypt. All the identified compounds had a characteristic peak at *m*/*z* 287 in the positive ionization mode (*m*/*z* 285 in the negative ionization mode) representing the cyanidin and/or kaempferol aglycone, which is further glycosylated with different sugars and/or acids (Table 1). In addition to the characteristic peak at *m*/*z* 285, compounds (A_1_–A_9_) showed both [M − 2H + H_2_O]^–^ and [M − 2H]^–^ doublet ions in the negative ionization mode, so they were identified as anthocyanins [7]. All detected anthocyanins (except for A_7_) mainly consisted of the cyanidin-3-glucoside, which is further acylated in different ways.

Compound A_1_ had [M]^+^ *m*/*z* 773 yielding MS/MS ions of *m*/*z* 611 [M-glucose-H_2_O]^+^, 449 [611-glucose-H_2_O]^+^, and 287 [449-glucose-H_2_O]^+^ and was tentatively identified as cyanidin-3-sophoroside-5-glucoside [1,6]. Compound A_2_ had [M]^+^ *m*/*z* 611 yielding MS/MS ions of *m*/*z* 449 [M-glucose-H_2_O]^+^ and 287 [449-glucose-H_2_O]^+^ and was tentatively identified as cyanidin-3, 5-diglucoside [1,6]. Compound A_3_ had [M]^+^ *m*/*z* 449 yielding MS/MS ions of *m*/*z* 287 [M-glucose-H_2_O]^+^ and was tentatively identified as cyanidin-3-*O*-glucoside [1,8,9,10].

Compounds A_4_, A_5_, and A_6_ were acylated with coumaric, ferulic, and sinapic acids. Compound A_4_ was tentatively identified as cyanidin-3-(p-coumaroyl)-sophoroside-5-glucoside. When [8] tabulated their results concerning the red cabbage cultivated in Sweden, they mistakenly identified an anthocyanin with a molecular weight of *m*/*z* 919 and a similar fragmentation pattern to that of compound A_4_ as cyan-3-(p-coumaroyl)-glucoside-5-glucoside, but according to the molecular weight of this compound (*m*/*z* 919) and its MS/MS data—*m*/*z* 757 [M-glucose-H_2_O]^+^, 449 [757-glucose-coumaroyl-(2 H_2_O)]^+^, and 287 [449-glucose]^+^—it should have been identified as cyanidin-3-(p-coumaroyl)-sophoroside-5-glucoside [6].

Compound A_5_ had [M]^+^ *m*/*z* 949 yielding MS/MS ions of *m*/*z* 787 [M-glucose-H_2_O]^+^, 449 [787-glucose-ferulic-(2 H_2_O)]^+^, and 287 [449-glucose-H_2_O]^+^ and was tentatively identified as cyanidin-3-(feruloyl)-sophoroside-5-glucoside [5,6]. Compound A_6_ had [M]^+^ *m*/*z* 979 yielding MS/MS ions of *m*/*z* 817 [M-glucose-H_2_O]^+^, 449 [817-glucose-sinapic- (2 H_2_O)]^+^, and 287 [449-glucose-H_2_O]^+^ and was tentatively identified as cyanidin-3-(sinapoyl)-sophoroside-5-glucoside [1,6,8,9,10].

**Figure 1:**
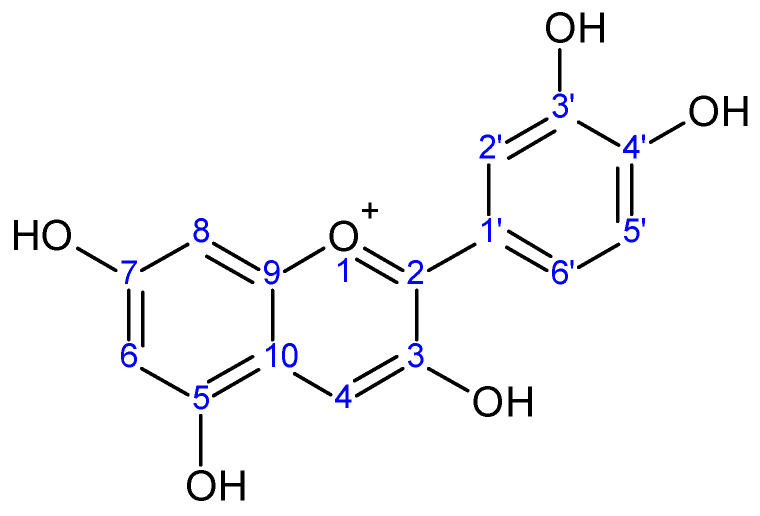
Cyanidin aglycone.

As for compound A_7_, it had [M]^+^ *m*/*z* 787 yielding MS/MS ions of *m*/*z* 625 [M-hexose]^+^, 449 [M-338]^+^, and 287 [M-500]^+^. Although the phytochemical dictionary of natural products database showed the previous identification of cyanidin-3-feruloyl-glucosyl-galactoside (glycosylation and acylation at C_3_ only), the retention data indicated that the glycosylation occurs at both C_3_ and C_5_. This is because an anthocyanin glycosylated and/or acylated at C_3_ only would have a free C_5_, and consequently an earlier retention time than that of A_7_ (Figure 1). Thus, compound A_7_ was tentatively identified as cyanidin-3-(feruloyl)-5-glucoside [6].

Compound A_8_ had [M]^+^ *m*/*z* 817 yielding MS/MS ions of *m*/*z* 655 [M-glucose-H_2_O]^+^, 449 [655-glucose-sinapic-(2 H_2_O)]^+^, and 287 [449-glucose-H_2_O]^+^ and was tentatively identified as cyanidin-3-(sinapoyl)-glucoside-5-glucoside, while compound A_9_ had [M]^+^ *m*/*z* 757 yielding MS/MS ions of *m*/*z* 595 [M-glucose-H_2_O]^+^ and 287 [595-glucose-rhamnose-(2 H_2_O)]^+^ and was tentatively identified as cyanidin-3-[2-glucosyl-6-rhamnosyl-glucoside] [1,6,9].

For compounds K_1_–K_5_, the anthocyanins characteristic [M − 2H + H_2_O]^−^ and [M − 2H]^−^ doublet ions were absent in the negative ionization mode, so they were tentatively identified as kaempferol derivatives. All these kaempferol derivatives were tentatively identified for the first time in the red cabbage leaves by using the high resolution UPLC-MS data (accurate mass and MS/MS fragmentation patterns).

Compound K_1_ had [M − H] *m*/*z* 803 yielding MS/MS ions of *m*/*z* 641 [M-glucose-H_2_O]^+^, 447 [641-hydroxyferuloyl-H_2_O]^+^, and 285 [447-glucose-H_2_O]^+^ and was tentatively identified as kaempferol-3-(hydroxyferuloyl)-glucoside-7-glucoside. Compound K_2_ had [M − H] *m*/*z* 935 yielding MS/MS ions of *m*/*z* 773 [M-glucose-H_2_O]^+^, 609 [773-hydroxycoumaroyl-H_2_O]^+^, 447 [609-glucose-H_2_O]^+^, and 285 [447-glucose-H_2_O]^+^ and was tentatively identified as kaempferol-3-(hydroxycoumaroyl)-sophoroside-7-glucoside. Compound K_3_ had [M − H] *m*/*z* 965 yielding MS/MS ions of *m*/*z* 803 [M-glucose-H_2_O]^+^, 447 [803-glucose-hydroxyferuloyl- (2 H_2_O)]^+^, and 285 [447-glucose-H_2_O]^+^ and was tentatively identified as kaempferol-3-(hydroxyferuloyl)-sophoroside-7-glucoside. Compound K_4_ had [M − H] *m*/*z* 995 yielding MS/MS ions of *m*/*z* 833 [M-glucose-H_2_O]^+^, 447 [833-glucose-hydroxysinapoyl-(2 H_2_O)]^+^, and 285 [447-glucose-H_2_O]^+^ and was tentatively identified as kaempferol-3-(hydroxysinapoyl)-sophoroside-7-glucoside. Compound K_5_ had [M − H] *m*/*z* 1183 yielding MS/MS ions of *m*/*z* 1021 [M-glucose-H_2_O]^+^ and 815 [1021-sinapoyl-H_2_O]^+^ and was tentatively identified as kaempferol-3-(disinapoyl)-sophoroside-7-glucoside.

All the isolated compounds (A_1_–A_9_ and K_1_–K_5_) are compiled in Table 1, with their retention time data, mass, tandem mass, relative degree of bond unsaturation (RDB), λ_acyl_, E_acyl_/E_vis_, λ_vis_, E_440_/E_vis_, and tentative identification.

### 3.2. Antioxidant Activity

The total antioxidant capacity (TAC) of the total alcoholic extract and the phenolic extract of red cabbage leaves was investigated using the FRAP assay. The phenolic extract showed about 96% antioxidant activity compared to the ferrous sulfate heptahydrate standard, while the total alcoholic extract showed no significant antioxidant activity (Figure S3). This may be due to the decomposition of natural polyphenols and anthocyanins after air-drying.

### 3.3. Antimicrobial Activity

The antimicrobial activity of the total alcoholic extract and the phenolic extract of red cabbage leaves against *Aspergillus niger*, *Bacillus subtilis*, *Candida albicans*, *Escherichia coli*, *Pseudomonas aeruginosa*, and *Staphylococcus aureus* compared to standard ampicillin, streptomycin, and clotrimazole was determined (Figure S4).

A significant antimicrobial activity was detected for the phenolic extract against *Staphylococcus aureus*, *Bacillus subtilis*, and *Escherichia coli* with MICs of about 4 μg/mL, 5 μg/mL, and 5 μg/mL, respectively. Furthermore, it had moderate antimicrobial activity against *Candida albicans* and *Aspergillus niger* with an MIC of about 10 μg/mL, but had no effect against *Pseudomonas aeruginosa*. Once again, the total alcoholic extract showed no significant antimicrobial activity. The minimum inhibitory concentrations of the phenolic extract and the standards against the aforementioned organisms were determined and recorded in Table S1.

### 3.4. Anticancer Activity

The anticancer activities of the total alcoholic extract and the phenolic extract of red cabbage leaves (in comparison with standard doxorubicin) were investigated against the HeLa, MCF-7, and HepG-2 cell lines, and the results are depicted in Table 2.

**Figure 2:**
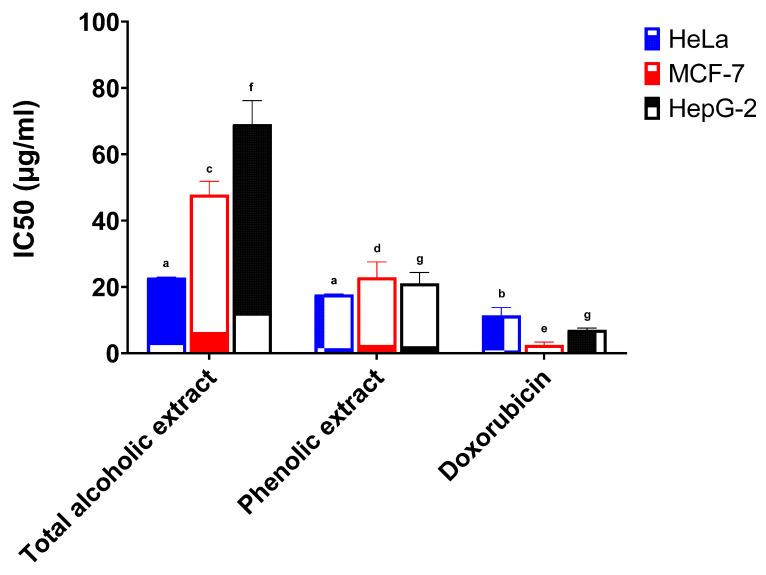
Anticancer activity of the total alcoholic extract and the phenolic extract of red cabbage leaves against HeLa, MCF-7, and HepG-2 cell lines. Bar graphs represent the mean ±SEM of 3 determinations (different letters above each column indicate statistically significant differences).

The total alcoholic extract and the phenolic extract of red cabbage leaves showed a high potency against the HeLa cell line. The half inhibitory concentrations (IC_50_) were 22.78 μg/mL and 17.71 μg/mL, respectively, and results were found to be statistically significant when compared to the standard drug doxorubicin (IC_50_ 11.38 μg/mL) (Figure 2). The total alcoholic extract gave weak activity both against the MCF-7 cell line with 5.3% activity and the HepG-2 cell line with 10.2% activity (IC_50_ 47.84 μg/mL and 69.11 μg/mL, respectively) when compared to standard doxorubicin (Figure 2). The phenolic extract showed weak to moderate activity against the MCF-7 cell line with 11.0% activity and the HepG-2 cell line with 33.4% activity (IC_50_ 22.89 μg/mL and 21.08 μg/mL, respectively) in comparison with standard doxorubicin (Figure 2).

The IC_50_ of the total alcoholic extract and the phenolic extract against the HeLa and MCF-7 cell lines were found to be statistically significant when compared to doxorubicin. The total alcoholic extract also showed statistical significance against the HepG-2 cell line in comparison with the phenolic extract and/or the standard doxorubicin. In addition, both extracts showed statistical significance to each other against MCF-7, but no significance was present for the HeLa cell line. The statistical significance of cell growth inhibition was determined using six different concentrations (compared to doxorubicin) and is shown in Figures S5–S7.

## 4. Conclusions

Due to their rising importance in the phytotherapeutic fields, a profiling of the anthocyanins and phenolics of the leaves of red cabbage cultivated in Egypt was performed using high resolution UPLC-PDA-ESI-qTOF-MS in the present study. Nine anthocyanins were tentatively identified as cyanidin-3-sophoroside-5-glucoside; cyanidin-3,5-diglucoside; Cyanidin-3-*O*-glucoside; cyanidin-3-(p-coumaroyl)-sophoroside-5-glucoside; cyanidin-3-(feruloyl)-sophoroside-5-glucoside; cyanidin-3-(sinapoyl)-sophoroside-5-glucoside; cyanidin-3-(feruloyl)-5-glucoside; cyanidin-3-(sinapoyl)-glucoside-5-glucoside; and cyanidin-3-[2-glucosyl-6-rhamnosyl-glucoside].

Five kaempferol derivatives were tentatively identified for the first time in the red cabbage leaves as kaempferol-3-(hydroxyferuloyl)-glucoside-7-glucoside, kaempferol-3-(hydroxycoumaroyl)-sophoroside-7-glucoside, kaempferol-3-(hydroxyferuloyl)-sophoroside-7-glucoside, kaempferol-3-(hydroxysinapoyl)-sophoroside-7-glucoside, and kaempferol-3-(disinapoyl)-sophoroside-7-glucoside.

The bioactivity assessment in the aforementioned therapeutic fields was conducted and showed that the phenolic extract of red cabbage leaves is efficient as an antioxidant; with 96% activity when compared to the ferrous sulfate heptahydrate standard, and had a significant antimicrobial activity against *Staphylococcus aureus*, *Bacillus subtilis*, and *Escherichia coli*, but had only a moderate effect against *Aspergillus niger* and *Candida albicans*, and no effect against *Pseudomonas aeruginosa*. Moreover, it had a very significant anticancer activity, especially against the human cervical (HeLa) cell line. Further studies are recommended to explore its possible future use in the aforementioned medical fields.

## Figures and Tables

**Table 1:** Anthocyanins and phenolics tentatively identified in the *Brassica oleracea* L. var. *capitata* f. *rubra* DC (red cabbage) leaves using high resolution UPLC-PDA-MS/MS in positive and negative ionization mode.

	Retention Time (min)	Mass [M^+^] or [M − H] ^a^	Tandem Mass	RDB ^b^	Error (ppm)	Λ_acyl_^c^	λ_vis_^d^	E_acyl_/E_vis_^e^	E_440_/E_vis_^f^	Formula	Tentative Identification
A_1_	1.35	773.2129	611.1597 449.1072 287.0548	13.5	−0.769		514		60%	C_33_H_41_O_21_	Cyanidin-3-sophoroside-5-glucoside
A_2_	1.53	611.1596	449.1073 287.0549	12.5	−1.720		515		41%	C_27_H_31_O_16_	Cyanidin-3,5-diglucoside
A_3_	8.65	449.1071	287.0551	11.5	−1.532		517		53%	C_21_H_21_O_11_	Cyanidin-3-O-glucoside
A_4_	10.56	919.2498	757.1967 449.1077 287.0551	19.5	−0.483	297	523	82%	43%	C_42_H_47_O_23_	Cyanidin-3-(p-coumaroyl)-sophoroside-5-glucoside
A_5_	10.67	949.2607	787.2076 449.1079 287.0553	19.5	0.486	295	523	123%	35%	C_43_H_49_O_24_	Cyanidin-3-(feruloyl)-sophoroside-5-glucoside
A_6_	10.79	979.2710	817.2170 449.1073 287.0549	19.5	−1.157	286	523	90%	34%	C_44_H_51_O_25_	Cyanidin-3-(sinapoyl)-sophoroside-5-glucoside
A_7_	10.82	787.2070	625.1549 449.1087 287.0552	18.5	−1.315	284	529	45%	50%	C_37_H_39_O_19_	Cyanidin-3-(feruloyl)-5-glucoside
A_8_	10.86	817.2183	655.1656 449.1080 287.0553	18.5	−0.306	284	529	41%	51%	C_38_H_41_O_20_	Cyanidin-3(sinapoyl)-glucoside-5-glucoside
A_9_	11.08	757.1954	595.1447 287.0552	18.5	−2.734		532		47%	C_36_H_37_O_18_	Cyanidin-3-[2-glucosyl-6-rhamnosyl-glucoside]
K_1_	9.89	803.2020	641.1506 447.0939 285.0403	18.5	−1.182	220	524	500%	51%	C_37_H_39_O_20_	Kaempferol-3-(hydroxyferuloyl)-glucoside-7-glucoside
K_2_	9.71	935.2445	773.1925 609.1457 447.0928 285.0404	19.5	−0.715	218	523	416%	39%	C_42_H_47_O_24_	Kaempferol-3-(hydroxycoumaroyl)-sophoroside-7-glucoside
K_3_	9.78	965.2549	803.2029 447.0929 285.0400	19.5	−0.894	218	523	384%	35%	C_43_H_49_O_25_	Kaempferol-3-(hydroxyferuloyl)-sophoroside-7-glucoside
K_4_	9.88	995.2654	833.2131 447.0914 285.0408	19.5	−0.932	219	524	476%	44%	C_44_H_51_O_26_	Kaempferol-3-(hydroxysinapoyl)-sophoroside-7-glucoside
K_5_	10.16	1183.3116	1021.2600 815.2025	26.5	−1.692	221	531	588%	57%	C_55_H_59_O_29_	Kaempferol-3-(disinapoyl)-sophoroside-7-glucoside

^a^ [M^+^] for the anthocyanins (A_1_–A_9_) and [M − H] For the kaempferol derivatives (K_1_–K_5_). ^b^ RDB: Relative degree of bond unsaturation. ^c^ λ_acyl_: The absorption maxima due to the chromophore of the acylating aromatic group at 286 nm. ^d^ λ_vis_: The absorption maxima due to the presence of the anthocyanidin chromophore at 520 nm. ^e^ E_vis_ and E_acyl_: The absorptivities at the corresponding maxima. ^f^ E440: The absorptivity at 440 nm.

**Table 2:** Half inhibitory concentrations (IC_50_) of the total alcoholic extract and the phenolic extract of red cabbage leaves as well as the standard doxorubicin against HeLa, MCF-7, and HepG-2 cell lines.

Extract	Anticancer Activity		
	HeLa IC_50_ ± SD (µg/mL)	MCF-7 IC_50_ ± SD (µg/mL)	HepG-2 IC_50_ ± SD (µg/mL)
Media/DMSO (–ve control)	NA	NA	NA
Total alcoholic extract	22.78 ± 0.39	47.84 ± 7.03	69.11 ± 12.29
Phenolic extract	17.71 ± 0.33	22.89 ± 8.09	21.08 ± 5.72
Doxorubicin	11.38 ± 4.17	2.52 ± 1.54	7.05 ± 1.01

## Data Availability

The data presented in this study is available in this article and Supplementary Materials.

## Supplementary Materials

The following are available online at: Table S1. Minimum inhibitory concentration (μg/mL) of the total alcoholic extract and the phenolic extract of red cabbage leaves against *Aspergillus niger*, *Bacillus subtilis*, *Candida albicans*, *Escherichia coli*, *Pseudomonas aeruginosa*, and *Staphylococcus aureus*. Figure S1. UPLC-MS chromatogram (positive ESI) of the red cabbage phenolic extract. Figure S2. UPLC-MS chromatogram (negative ESI) of the red cabbage phenolic extract. Figure S3. Total antioxidant capacity (TAC) of the total alcoholic extract and the phenolic extract of red cabbage leaves calculated for 4 min at 593 nm. Figure S4. Antimicrobial activity of the total alcoholic extract and the phenolic extract of red cabbage leaves against *Staphylococcus aureus, Bacillus subtilis, Escherichia coli, Pseudomonas aeruginosa, Candida albicans and Aspergillus niger* compared to standard Ampicillin, Streptomycin and Clotrimazole. The phenolic extract and all the standards were prepared as 100 μg/mL. Figure S5. Anticancer activity of various concentrations of the total alcoholic extract and the phenolic extract of red cabbage leaves against the cervical cancer cell line; HeLa. Figure S6. Anticancer activity of various concentrations of the total alcoholic extract and the phenolic extract of red cabbage leaves against the breast cancer cell line; MCF-7. Figure S7. Anticancer activity of various concentrations of the total alcoholic extract and the phenolic extract of red cabbage leaves against the liver cancer cell line; HepG-2.

## Sample Availability

Samples are available from the authors.
