# Supplementary material for: High-Resolution UPLC-MS Profiling of Anthocyanins and Flavonols of Red Cabbage (Brassica oleracea L. var. capitata f. rubra DC.) Cultivated in Egypt and Evaluation of Their Biological Activity
Source: Molecules. 2021 Dec 14;26(24):7567. doi: 10.3390/molecules26247567 (PMC8708035; doi:10.3390/molecules26247567)
Supplement: Supplementary file 1 [file molecules-26-07567-s001.zip › molecules-1505963-supplementary.pdf]

molecules

MDPI

Supplementary Materials

High-Resolution UPLC-MS Profiling of Anthocyanins and Flavonols of Red Cabbage (*Brassica oleracea* L. var. *capitata* f. *rubra* DC.) Cultivated in Egypt and Evaluation of their Biological Activity

Khaled Ahmed Mansour [1,2,*], Sherifa Fahmy Moustafa [1,3] and Soad Mohamed Abdelkhalik [4]

1 Pharmacognosy Department, Faculty of Pharmacy, October 6 University, Al Mehwar Al Markazi, Giza P.O. Box 12585, Egypt; sherifa.mostafa@pharma.cu.edu.eg
2 Pharmacognosy Department, Faculty of Pharmacy, Horus University in Egypt, New Damietta P.O. Box 34517, Egypt
3 Pharmacognosy Department, Faculty of Pharmacy, Cairo University, Kasr el Aini st., Cairo P.O. Box 11562, Egypt
4 Pharmacognosy Department, Faculty of Pharmacy, Helwan University, Ain-Helwan, Cairo P.O. Box 11795, Egypt; soad_abdelkhalik@pharm.helwan.edu.eg
* Correspondence: khaled.mansour@o6u.edu.eg; Tel.: +20-010-98-424-801

Table S1. Minimum inhibitory concentration (µg/mL) of the total alcoholic extract and the phenolic extract of red cabbage leaves against Aspergillus niger, Bacillus subtilis, Candida albicans, Escherichia coli, Pseudomonas aeruginosa, and Staphylococcus aureus.

	MIC (µg/mL)					
	Staphylococcus aureus	***Bacillus subtilis***	***Escherichia coli***	***Pseudomonas aeruginosa***	***Candida albicans***	***Aspergillus niger***
**Total alcoholic extract**	**0**	**0**	**0**	**0**	**0**	**0**
**Phenolic extract**	**4**	**5**	**5**	**0**	**10**	**10**
**Ampicillin**	**1**	**5**	——	——	——	——
**Streptomycin**	——	——	**1**	**5**	——	——
**Clotrimazole**	——	——	——	——	**2**	**10**

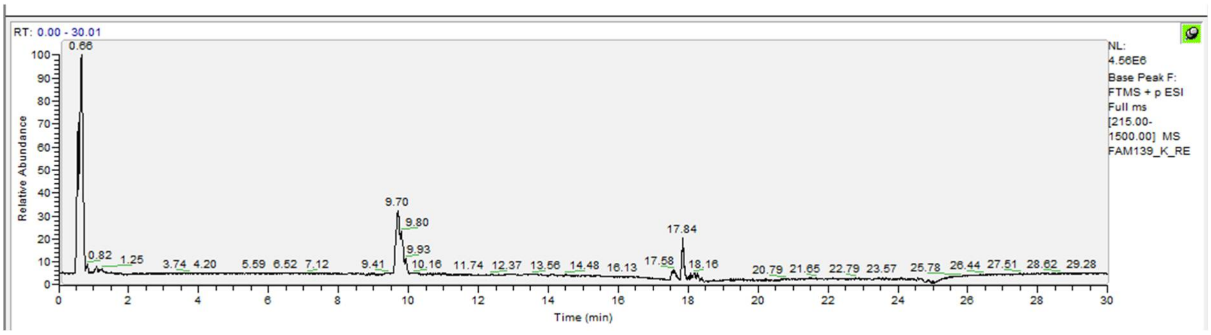

Figure S1. UPLC-MS chromatogram (positive ESI) of the red cabbage phenolic extract.

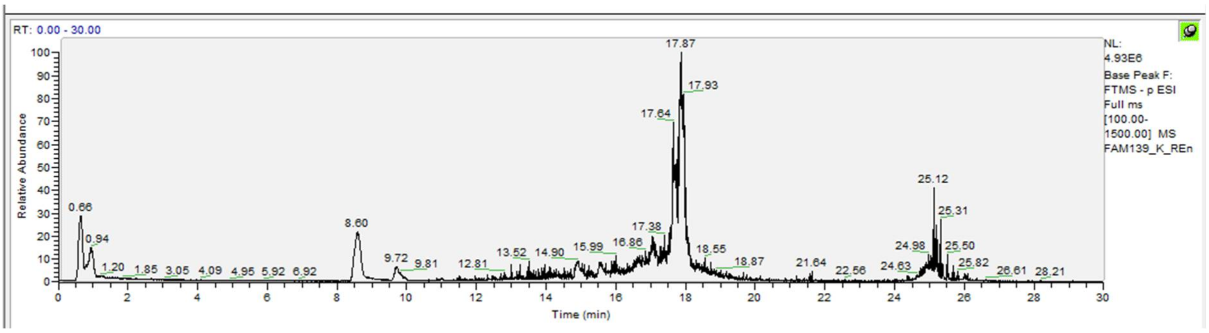

Figure S2. UPLC-MS chromatogram (negative ESI) of the red cabbage phenolic extract.

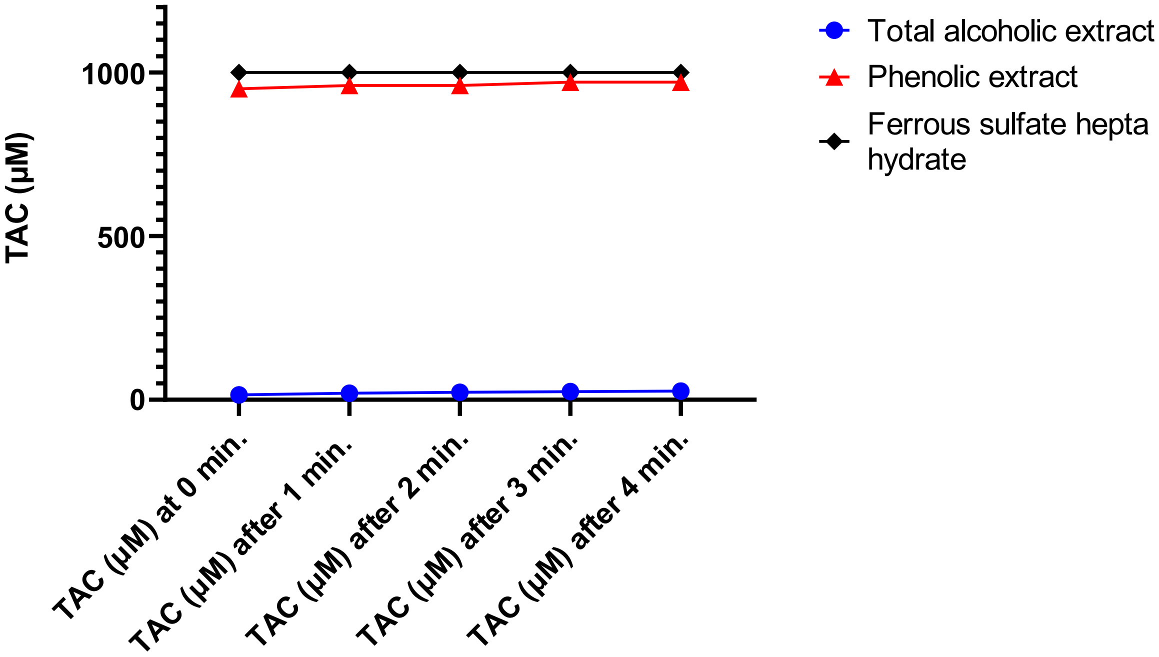

Figure S3. Total antioxidant capacity (TAC) of the total alcoholic extract and the phenolic extract of red cabbage leaves calculated for 4 minutes at 593 nm.

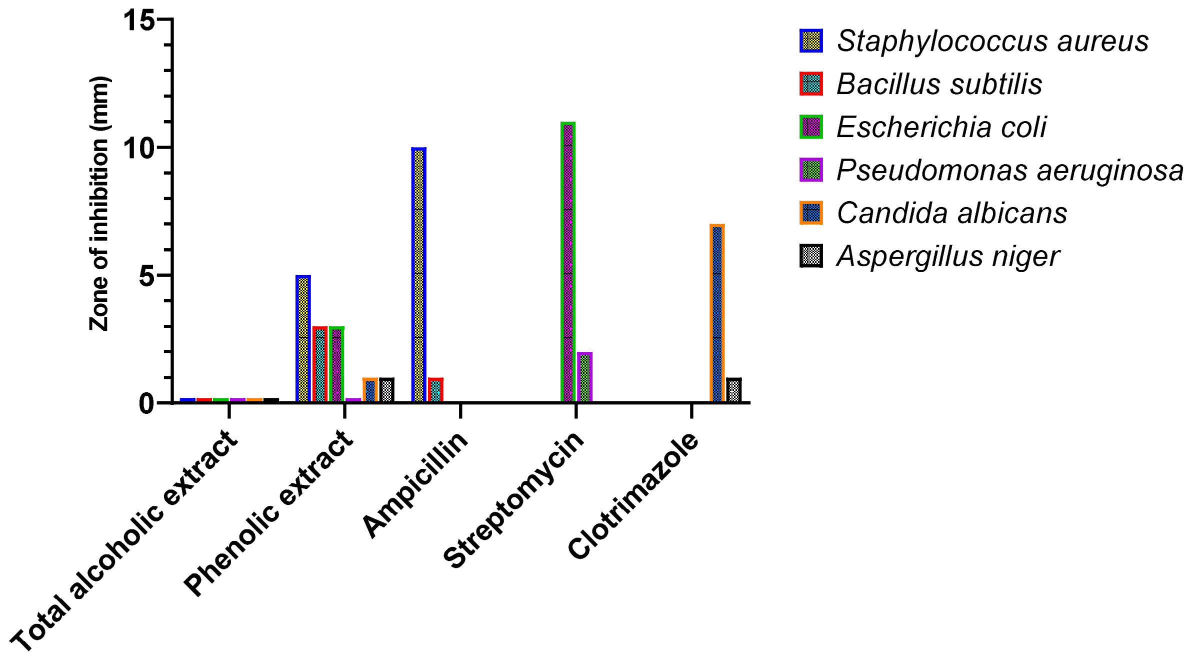

Figure S4. Antimicrobial activity of the total alcoholic extract and the phenolic extract of red cabbage leaves against *Staphylococcus aureus, Bacillus subtilis, Escherichia coli, Pseudomonas aeruginosa, Candida albicans and Aspergillus niger* compared to standard Ampicillin, Streptomycin and Clotrimazole. The phenolic extract and all the standards were prepared as 100 µg/mL.

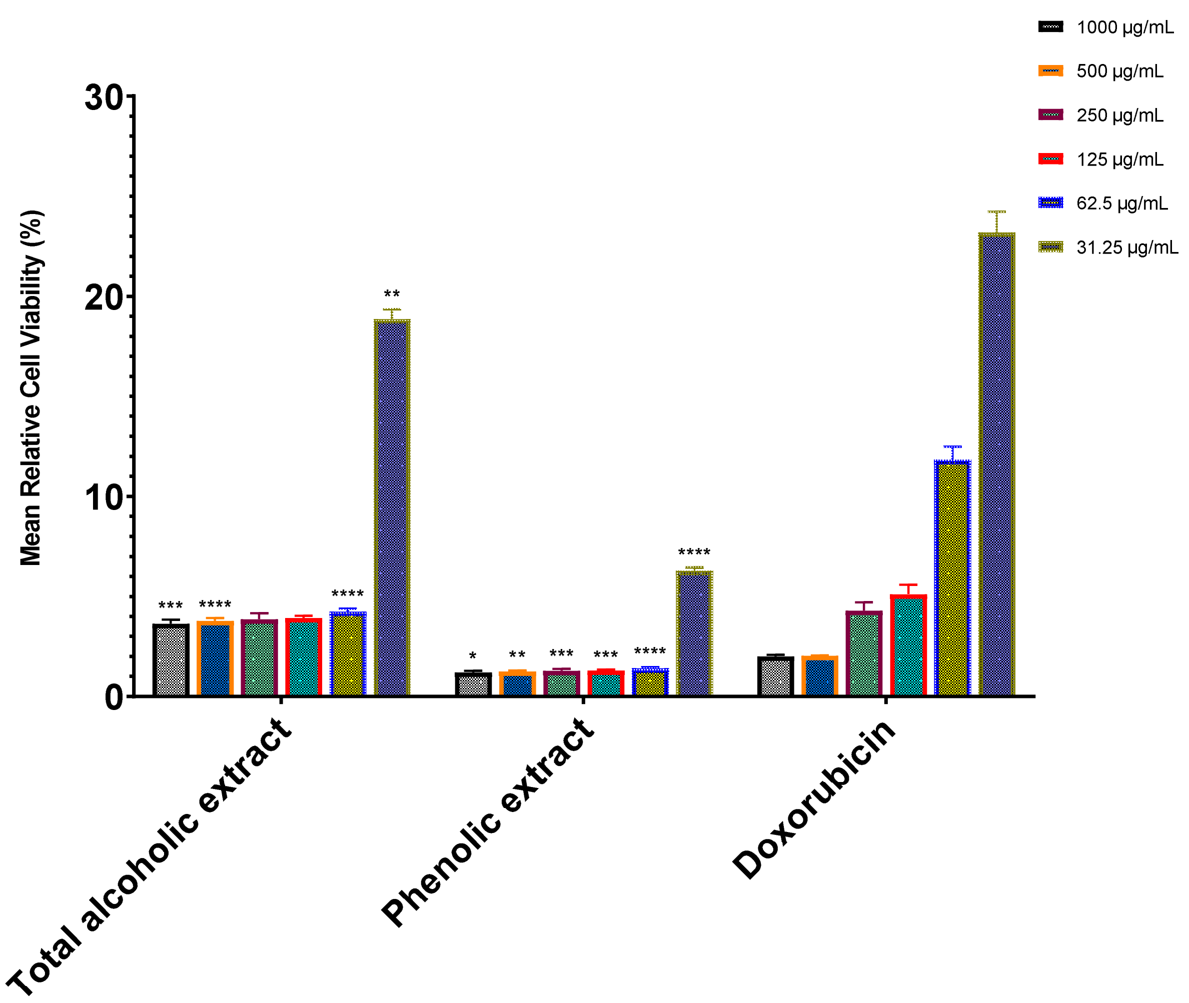

Figure S5. Anticancer activity of various concentrations of the total alcoholic extract and the phenolic extract of red cabbage leaves against the cervical cancer cell line; HeLa. Bar graphs represent the mean ± SEM of 3 determinations. Asterisks show statistical significance ($p \leq 0.05$) compared to the reference drug doxorubicin. (*) denotes $p \leq 0.05$. (**) denotes $p \leq 0.01$. (***) denotes $p \leq 0.001$. (****) denotes $p \leq 0.0001$.

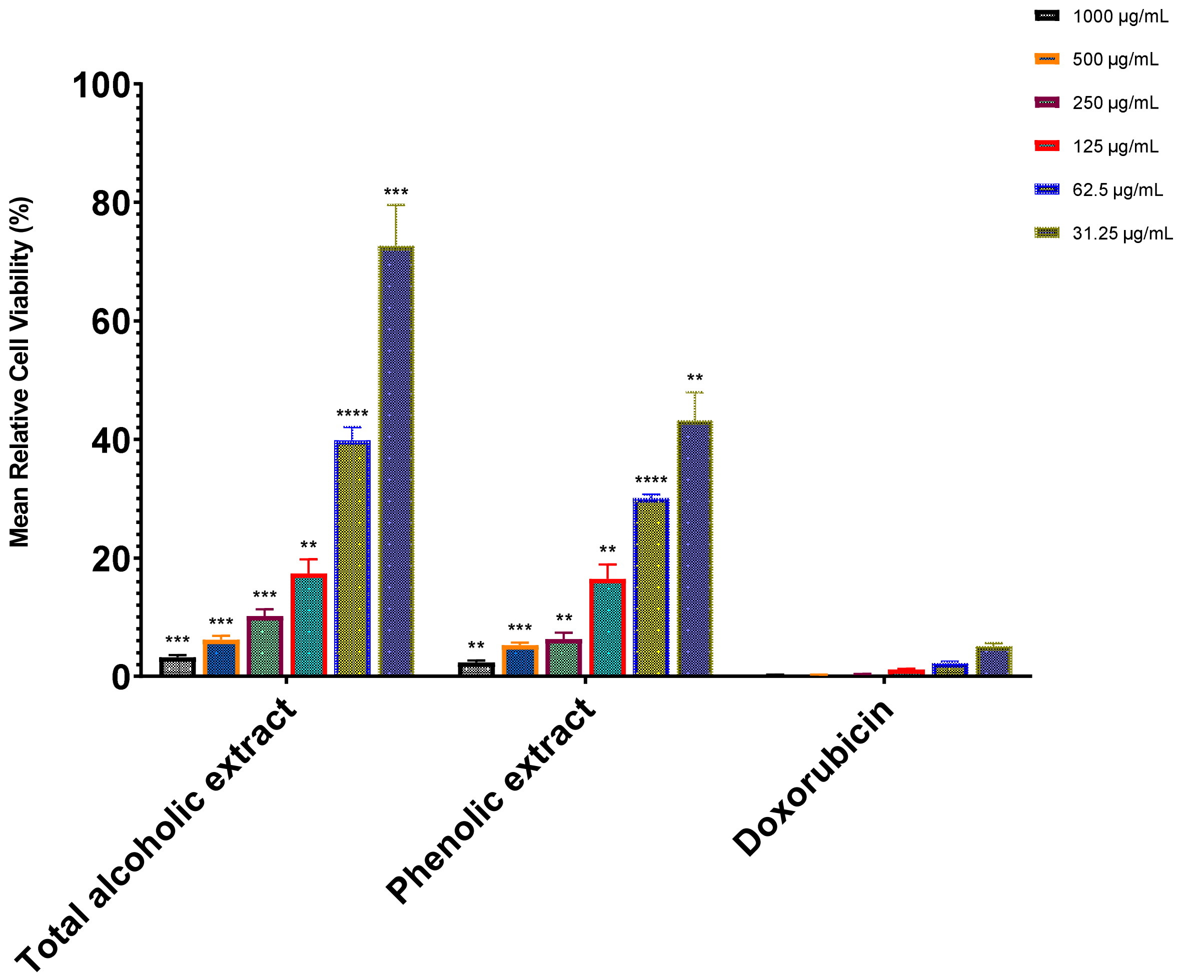

Figure S6. Anticancer activity of various concentrations of the total alcoholic extract and the phenolic extract of red cabbage leaves against the breast cancer cell line; MCF-7. Bar graphs represent the mean ± SEM of 3 determinations. Asterisks show statistical significance ($p \leq 0.05$) compared to the reference drug doxorubicin. (*) denotes $p \leq 0.05$. (**) denotes $p \leq 0.01$. (***) denotes $p \leq 0.001$. (****) denotes $p \leq 0.0001$.

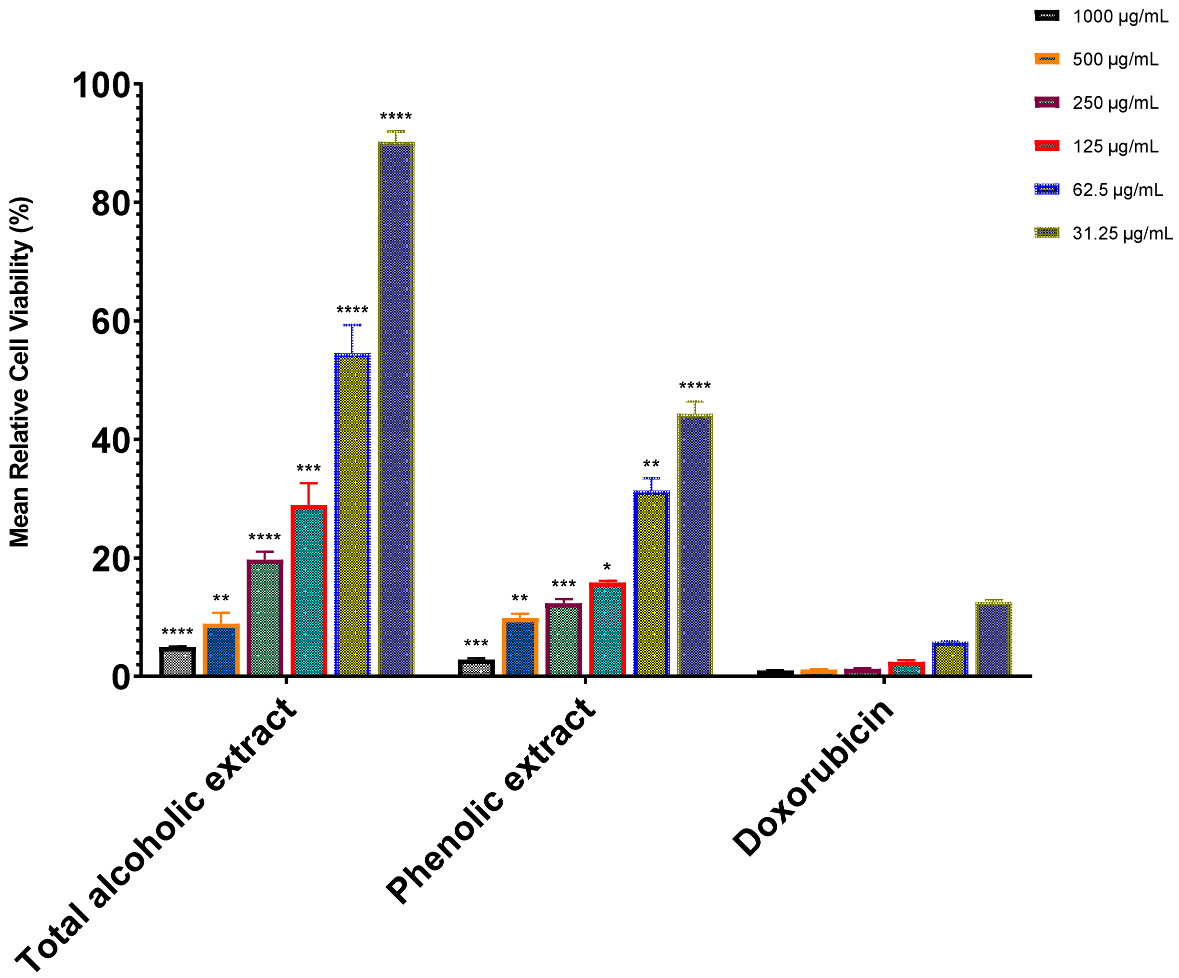

Figure S7. Anticancer activity of various concentrations of the total alcoholic extract and the phenolic extract of red cabbage leaves against the liver cancer cell line; HepG-2. Bar graphs represent the mean ± SEM of 3 determinations. Asterisks show statistical significance ($p \leq 0.05$) compared to the reference drug doxorubicin. (*) denotes $p \leq 0.05$. (**) denotes $p \leq 0.01$. (***) denotes $p \leq 0.001$. (****) denotes $p \leq 0.0001$.
